# Should continuous glucose monitoring be used to manage neonates at risk of hypoglycaemia?

**DOI:** 10.3389/fped.2023.1115228

**Published:** 2023-03-21

**Authors:** Maria-Sofia Kalogeropoulou, Isabel Iglesias-Platas, Kathryn Beardsall

**Affiliations:** ^1^ School of Clinical Medicine, University of Cambridge, Cambridge, United Kingdom; ^2^ Department of Paediatrics, Norfolk and Norwich University Hospital, Norwich, United Kingdom; ^3^ Department of Paediatrics, School of Clinical Medicine, University of Cambridge, Cambridge, United Kingdom; ^4^ Neonatal Intensive Care Unit, Rosie Hospital, Cambridge University Hospitals NHS Foundation Trust, Cambridge, United Kingdom

**Keywords:** continuous glucose monitor (CGM), neonatal care, hypoglycaemia, dexcom, medtronic

## Abstract

The National Institute for Clinical Excellence (NICE) now recommends that continuous glucose monitoring (CGM) be offered to adults and children with diabetes who are at risk from hypoglycaemia. Hypoglycaemia is common in the neonatal period, and is a preventable cause of poor neurodevelopmental outcome, but is CGM helpful in the management of neonates at risk of hypoglycaemia? Neonatal studies have shown that CGM can detect clinically silent hypoglycaemia, which has been associated with reduced executive and visual function in early childhood. Intervention trials have further shown CGM can support the targeting of glucose levels in high-risk extremely preterm neonates. In spite of significant advances in technology, including smaller sensors, better accuracy and factory calibration, further progress and adoption into clinical practice has been limited as current devices are not designed nor have regulatory approval for the specific needs of the newborn. The use of CGM has the potential to support clinical management, and prevention of hypoglycaemia but must be set within its current limitations. The data CGM provides however also provides an important opportunity to improve our understanding of potential risks of hypoglycaemia and the impact of clinical interventions to prevent it.

## Introduction

Following early controversies regarding the use of continuous glucose monitoring (CGM) in patients with diabetes, it has now become standard of care for many patients. Recent NICE guidelines recommend that all adults and children with type 1 diabetes should have access to CGM, along with some patients with type 2 diabetes who use insulin or experience recurrent or severe hypoglycaemia (1). Hypoglycaemia is known to be common in neonates with some infants considered at increased risk due to impaired metabolic regulation (2, 3). Research studies using masked CGM have highlighted significant periods of clinically silent exposure in both preterm (4, 5) and term neonates (6, 7). This silent hypoglycaemia has been associated with worse executive function and visual acuity in early childhood (8).

Given that hypoglycaemia is a frequent and often persistent finding in the neonatal period the potential advantages of CGM for clinical care are not difficult to appreciate. There is potential for real-time CGM to prospectively prevent hypoglycaemia in preterm neonates (4, 9–11), those born to mothers with diabetes (12, 13), those affected by congenital hyperinsulinism (14, 15) or with hypoxic-ischemic encephalopathy (16). However, there are significant practical limitations as the equipment is not designed for neonatal use. The more recent CGM systems with shorter warm up times, smaller sensors, improved accuracy at lower glucose thresholds, no need for calibration and longer lifespan have substantially increased their applicability for neonates. The benefits appear promising but there are significant limitations of its use and challenges for future implementation. CGM is now being used by some neonatal teams as part of standard of care for infants at risk of dysglycaemia, particularly in prevention of hyperglycaemia, but should it be used to manage babies at risk of hypoglycaemia?

## What is continuous glucose monitoring?

**Figure 1:**
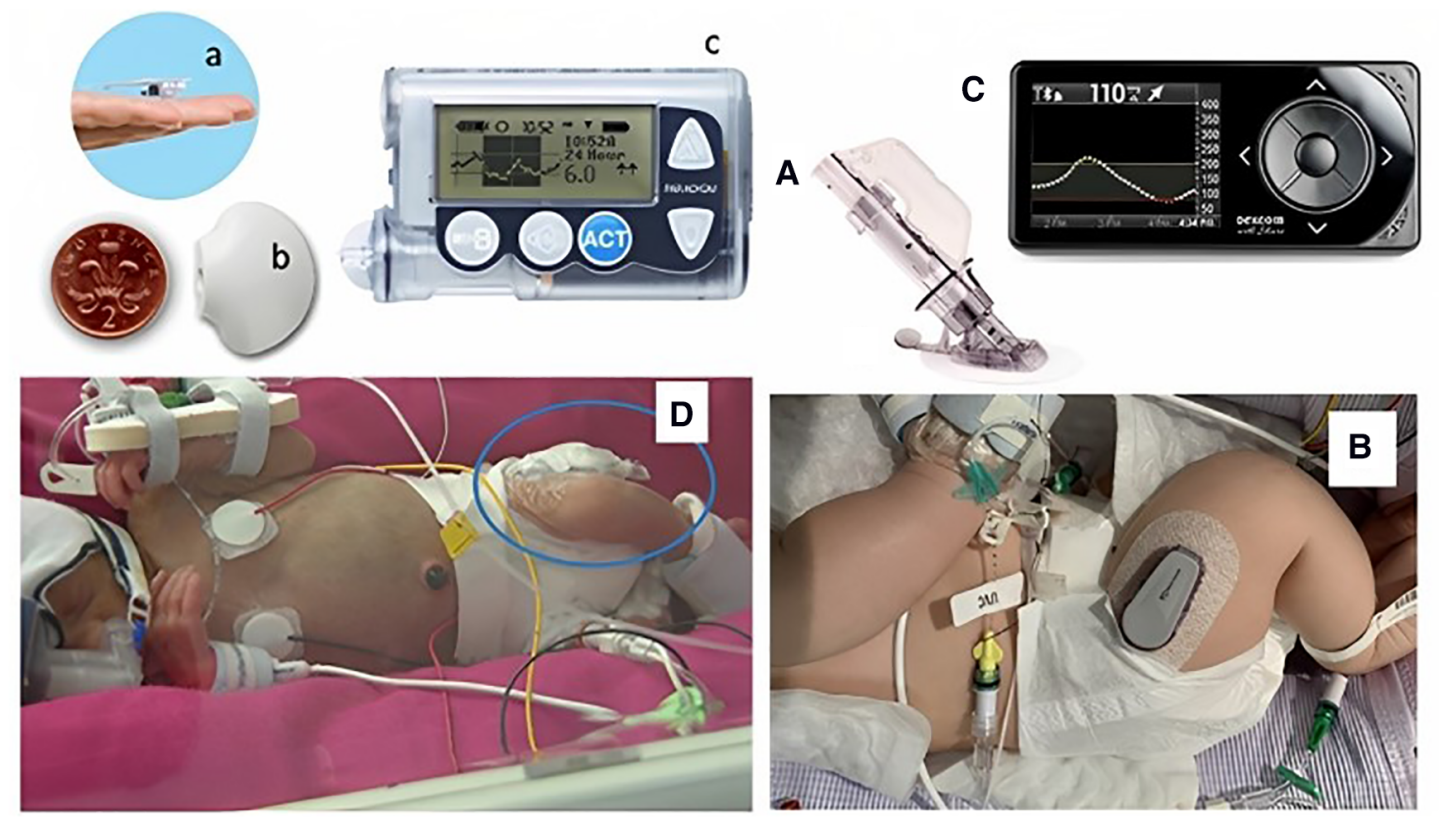
Continuous glucose monitoring systems *in situ* in two neonates. Neonates with continuous glucose monitoring *in situ*. Left panel showing Medtronic Guardian in a preterm baby: (**a**) sensor, (**b**) transmitter, (**c**) monitor, (**D**) baby with sensor in thigh. Right panel showing a Dexcom G4 with: (**A**) needle inserter, (**B**) transmitter on a baby's thigh, and (**C**) monitor. Figure reproduced from Myat Win, Rowan Beckett, Lynn Thomson, Ajay Thankamony, Kathryn Beardsall, continuous glucose monitoring in the management of neonates with persistent hypoglycemia and congenital hyperinsulinism, *The Journal of Clinical Endocrinology & Metabolism*, Volume 107, Issue 1, January 2022, Pages e246–e253, https://doi.org/10.1210/clinem/dgab601 under the terms of the creative commons CC BY license.

Continuous glucose monitoring systems consist of an interstitial glucose sensor, a transmitter, and a receiver that together provide the ability to have a continuous real time glucose measurement over a prolonged period (up to 10 days), without the need for repeated blood sampling. The sensor is a fine disposable filament that uses an oxidase-based platinum electrode, which catalyses interstitial glucose. This generates an electrical current, dependent on glucose concentration, which is transmitted to a monitor for real-time display and storage as a specific glucose value. Although measuring interstitial glucose, devices are calibrated to provide a value equivalent to the blood glucose concentration, either by factory settings, or through individual patient calibration with intermittent blood sampling. Although in adults the sensors can be placed on the abdominal wall and arms, in the neonate, with limited subcutaneous tissue, the sensor is usually inserted on the lateral aspect of the thigh (17). There are two main companies whose CGM systems (with a number of different models) have been used in neonates: Dexcom (SanDiego, CA, USA) and Medtronic (Northridge, CA, USA) (Figure 1). However, neither of them has been designed specifically for, or has obtained specific regulatory approval for use in the neonate.

## What are the potential benefits?

### An early warning system—reducing silent hypoglycaemia

In the setting of neonatal intensive care physiological parameters such as oxygen saturation and carbon dioxide are measured continuously, exposing trends that allow for a more proactive and safer management. Current strategies for glucose monitoring however, rely on intermittent blood sampling which is often done infrequently to minimise the stress of handling and blood loss, and consequently leaves prolonged periods between glucose measurements.

**Figure 2:**
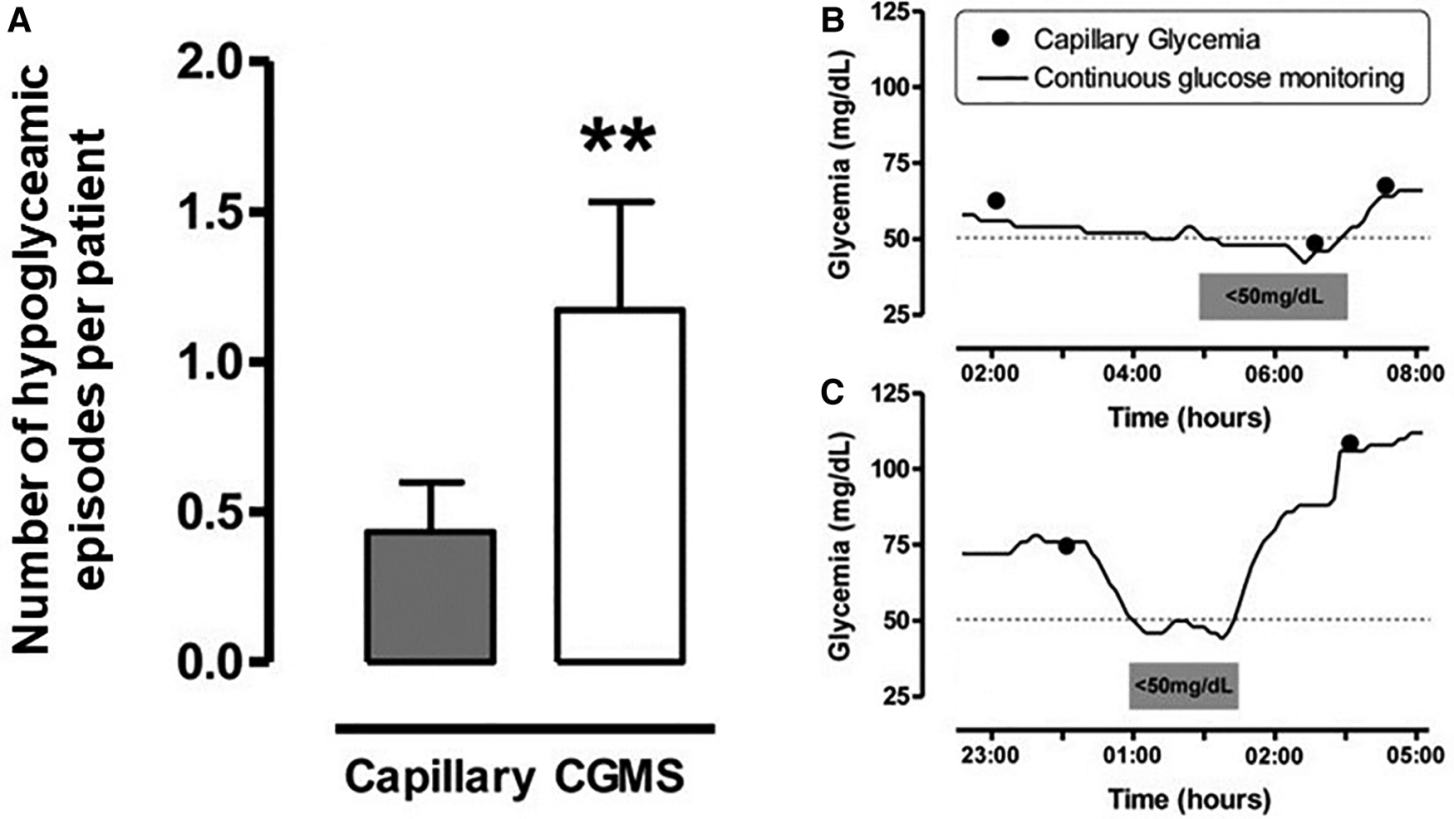
Number of Hypoglycaemia episodes per patient in those with CGM and examples of hypoglycaemic episodes Left panel (**A**): Number of hypoglycaemic episodes per patient detected by capillary blood glucose testing every 4 hours or from masked CGM data. Y-axis label modified from the reference. Hypoglycaemia was defined as <50mg/dl (2.8 mmol/L), number of episodes expressed as mean±SE *p* < 0.01 (Left panel). Right panel (**B**) and (**C**): Examples of hypoglycaemic episodes. Figure reproduced from Uettwiller F, Chemin A, Bonnemaison E, Favrais G, Saliba E, Labarthe F. Real-time continuous glucose monitoring reduces the duration of hypoglycemia episodes: a randomized trial in very low birth weight neonates. *PLoS ONE*. 2015 Jan 15;10(1):e0116255. doi: 10.1371/journal.pone.0116255. PMID: 25590334; PMCID: PMC4295867. Under the terms of the “https://creativecommons.org/licenses/”Creative Commons CC BY license.

CGM provides the clinical team with a continuous trend of glucose concentration over time, allowing early detection of falling levels and giving opportunities for early intervention. Alarms can be set at specific patient thresholds for hypoglycaemia, as well as trend alarms for rapid rates of change. It has been demonstrated in a range of neonates that masked CGM can identify clinically silent episodes of hypoglycaemia (Figure 2), and with real time monitoring these episodes could be pre-empted and prevented with appropriate management (10, 11, 13).

### Personalised medicine

Availability of continuous glucose values has also highlighted a high degree of variability of glucose levels in the population of sick neonates (11, 18). This combined with the frequent changes of drugs and infusion rates means that a one approach fits all does not work. This is an ever-growing challenge, as paediatricians are caring for increasingly preterm neonates in whom glucose control is even more variable. Current clinical practice relies on standard protocols for glucose control, and the use of insulin for the management of hyperglycaemia, leads to an increased risk of hypoglycaemia. In this setting CGM provides the opportunity for more personalised care and the potential for a platform for automatization that allows for intra and inter patient variability over time (19). This has the potential to result in increased patient safety, and a more efficient use of staff time (20).

### Reduced interventions

Uettwiller et al. showed that real-time CGM reduced the number of blood samples by 25% in very low birthweight neonates compared to standard practice with intermittent glucose sampling (10). Reduced interventions are associated with improved clinical outcomes, both due to limiting blood loss and the effect of pain from heel pricks (10, 21, 22). In fact, neonates have lower pain scores during CGM sensor insertion compared to heel stick blood samples (11). Given that once inserted the CGM system lasts up to 10 days, there is the potential to considerably reduce pain exposure over time, through more targeted and less frequent blood sampling.

## What types of neonates may benefit?

### Preterm

A number of studies using CGM in preterm neonates have shown that CGM can increase the time spent in the euglycaemic range (2.6–10 mmol/L, 47–180 mg.dl) (11, 23). The study by Thomson et al. showed per cent time in the target range (sensor glucose 2.6–10 mmol/L, 47–180 mg.dl) was greater with CGM than intermittent blood glucose measurement (77% vs. 59%, respectively) and it was highlighted that the CGM also detected clinically unsuspected episodes of hypoglycaemia (23). In the Galderisi study neonates in the unblinded CGM group had a greater percentage of time spent in euglycemic range (median, 84% vs. 68%, *p* < .001) and decreased time spent in mild (*p* = .04) and severe (*p* = .007) hypoglycaemia compared with the blinded CGM group (24). The study by Perri et al., showed that using CGM alarms for stricter target thresholds of glucose control (3.44–7.78 mmol/L, 62–140 mg/dl) combined with 33% dextrose infusion, as needed, compared to the use of CGM set to more conventional glucose thresholds (2.61–10 mmol/L, 47–180 mg/dl) resulted in significantly less dysglycaemia (24). This highlights the role of CGM in providing clinical teams with the information to allow them to be proactive in management and potentially prevent hypoglycaemia. In these preterm neonates CGM has also been shown to be cost effective even in the short time window of time to discharge (25).

CGM has been combined with artificial intelligence control algorithms to create closed-loop systems, guiding either glucose infusion (24) or insulin delivery (19). Galderisi et al. showed that real-time CGM alarms can be coupled with a computer-based, proportional-integrative-derivative control algorithm to calculate the glucose infusion required. Use of CGM plus the glucose infusion algorithm allowed for tighter control of the glucose concentration (*p* < 0.001), without affecting the neonates' growth (24). In contrast Beardsall et al. (19) reported the use of a closed-loop CGM-insulin delivery system in preterm neonates between 48 and 72 h of life. The time spent with glucose 4–8 mmol/L (72–144 mg/dl) was significantly increased with the use of the closed-loop system compared to manual insulin delivery (*p* < 0.001). Importantly, despite all neonates in the intervention arm receiving an insulin infusion, there were no hypoglycaemic episodes and there was no difference in the total insulin infused between study arms.

### Perioperative and transitioning onto feeds

**Figure 3:**
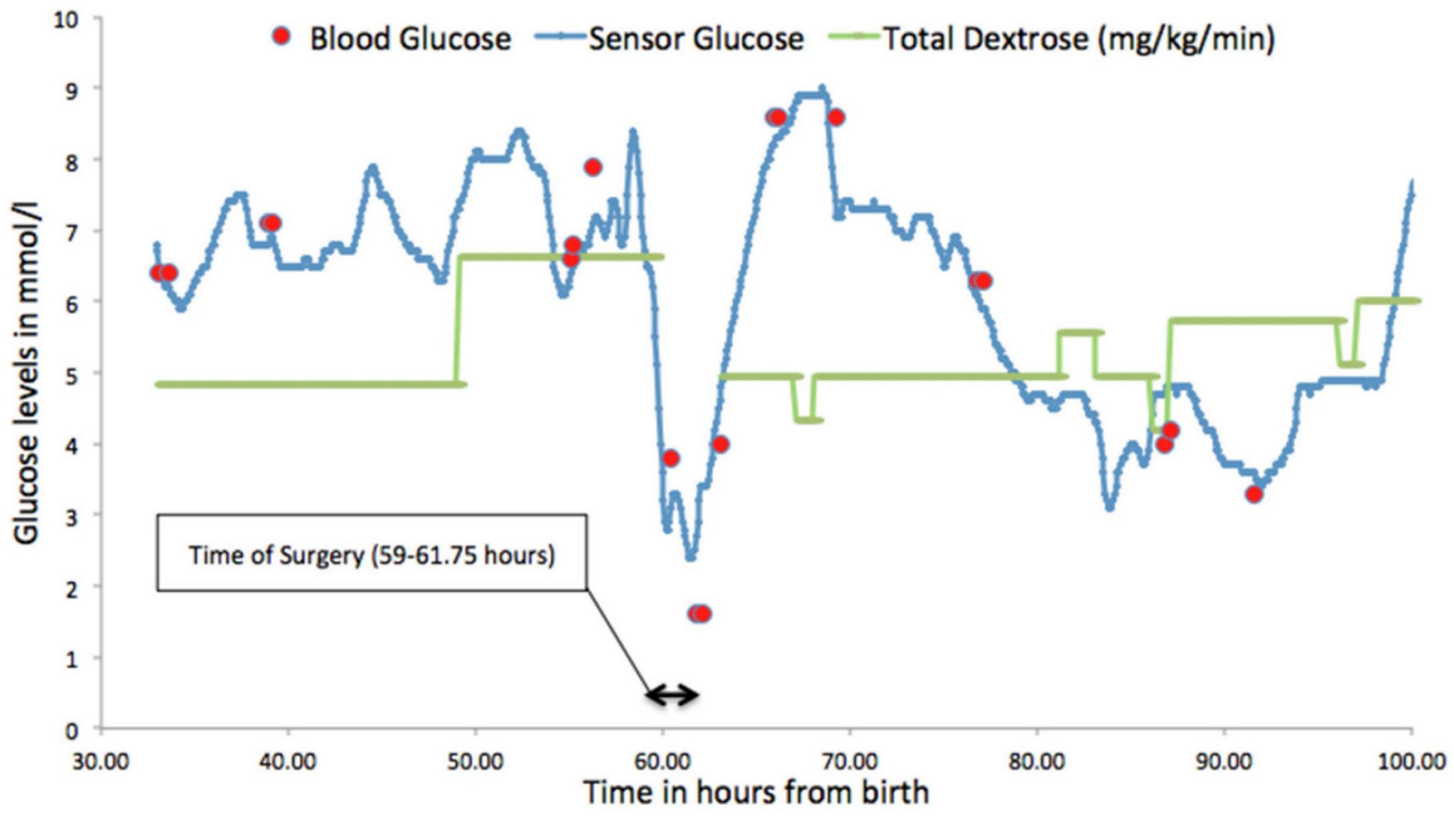
Glucose profile demonstrating fluctuating glucose levels in a neonate during surgery for necrotising enterocolitis. Figure reproduced with permission from Saha P, Beardsall K Perioperative continuous glucose monitoring in a preterm infant. *Case Reports* 2018;**2018:**bcr-2018–224728.

Figure 3 demonstrates the glucose profile of a preterm neonate undergoing surgery and highlights the potential benefits of CGM in this setting where monitoring can be difficult and changes in rates of fluid infusions can lead to rapid falls in glucose levels putting them at risk of hypoglycaemia (26). For preterm neonates who are presumed to be stable, tolerating intermittent feeds prior to going home, it has been shown that when monitored with CGM, they demonstrated significant dysglycaemia (5, 27). Forty percent of neonates born at < 1000 g, studied at corrected gestation age of 32 ± 2 and 33 ± 2 weeks, when considered clinically stable showed clinically silent hypoglycaemia [sensor glucose (SG) < 2.5 mmol/L, 45 mg/dl] (5).

### Congenital hyperinsulinism and persistent hypoglycaemia

CGM has also been used as an adjunct to clinical management of neonates with persistent hypoglycaemia and congenital hyperinsulinism (CHI) (15). This study highlighted the rapid fluctuations in glucose concentration in neonates with CHI which makes management with traditional intermittent blood sampling extremely challenging. These neonates are typically managed with hourly heel pricks which is distressing for both babies and carers. In a case study of a neonate with CHI, use of CGM prevented severe hypoglycaemia through the use of a personalised alert setting (14).

### Hypoxic ischaemic encephalopathy

Neonates with hypoxic ischaemic encephalopathy (HIE) are also at risk of hypoglycaemia which may compound ischaemic injury. Masked CGM has been used in this cohort and shown associations between dysglycaemia and worse neurodevelopmental outcomes (16, 28–30). Implementation of CGM use as part of clinical decision making in HIE has not been explored, and needs to be preceded by validation of sensor accuracy during cooling. A recent study shows that CGM accuracy is comparable in normothermic and hypothermic conditions (31).

### Late preterm and term neonates at risk

Studies using masked CGM in neonates of mothers with both type I and type II diabetes have uncovered significant periods of clinically silent hypoglycaemia. The CONCEPTT neonate study collected data on 16 mother and neonate dyads. Fifteen neonates had at least one blood glucose (BG) concentration < 47 mg/dl (2.6 mmol/L), and 4 neonates spent > 50% time with SG < 47 mg/dl (2.6 mmol/L) in the first 24 h after delivery, with a poor relationship between maternal intrapartum and neonatal glucose control (32). Interventional studies have not as yet been undertaken with this cohort of neonates.

Similarly large observational studies by the CHYLD team of term and late preterm neonates at risk of hypoglycaemia, using masked CGM, have shown an association between CGM-detected but clinically silent hypoglycaemia and worse neurocognitive outcomes at 4.5 years of age (8). This however was not associated with a significantly lower educational achievement at 9–10 years of age (33). These studies showed that despite regular BG testing and a clinical aim to maintain BG > 2.6 mmol/L (47 mg/dl), many neonates were exposed to prolonged periods when the SG was < 2.6 mmol/L. Furthermore, of the neonates who did develop hypoglycaemia (BG < 2.6 mmol/L, 47 mg/dl) 25% spent at least 5 h with SG < 2.6 mmol/L (47 mg/dl), and nearly a quarter of neonates who were considered to have normal BG levels by routine monitoring had episodes of clinically silent hypoglycaemia. Developments however are needed to provide diagnostic accuracy at the thresholds for hypoglycaemia in neonates which are significantly lower than those used in adults, to provide confidence in their use at this time (34). The potential to support proactive monitoring of such at-risk neonates to prevent hypoglycaemia vs. the potential to increase unnecessary interventions and maternal and neonate separation requires further evaluation.

## What are the device limitations for use in the neonate?

### Insertion

The manufacturers recommended methods of insertion and inserters for devices cannot be used in some neonates due to the limited subcutaneous tissue. In clinical studies hand insertion has been shown to be effective but requires specific training (4). Devices designed for neonatal insertion would significantly impact on the clinical utility of these systems.

### Warm up time

Although the “warm up” or “wetting time” for sensors has fallen dramatically in the last few years this means that glucose values are not immediately available to the clinical team (35). Furthermore, accuracy historically improved after the first 24 h after sensor insertion (36, 37). Possible explanations for this include a local inflammatory response and tissue microhaemorrhages post-insertion (38, 39). This potentially limits their use in the late preterm and term neonates who are considered most at risk in the first day after birth. Sensors specifically developed for neonates could potentially reduce the warm-up time and improve accuracy, by limiting the post-insertion inflammatory response.

### Point accuracy vs. trends

Point-accuracy compared to blood samples has historically been used as a primary criterion for regulatory approval for glucose monitors (40). It is measured as the mean absolute relative difference (MARD), and typically, a MARD < 10% is considered to indicate good performance. However, the reported MARD for CGM tends to be relatively high (8.7% to 18%) in neonatal studies (13, 41).

There are a number of physiological reasons for the differences in CGM values compared to the “gold standard” blood glucose values. Firstly CGM is measuring interstitial glucose not blood glucose and there is a well-known lag time between glucose levels in the blood and in the interstitial fluid (42). The diffusional speed is dependent on multiple factors, such as the blood glucose levels, the permeability of the tissue, the blood flow and the circulating insulin and glucagon hormones (43). Recent advances in CGM systems can reduce the lag to 2.1 ± 5.0 min for children (44). The main clinical issue arises during times of rapid change, with an increasing positive error during glucose drops meaning a “delay” in detecting hypoglycaemia. However this needs to be considered in the context of continuously available data in contrast to the relative infrequent blood glucose measurements.

Timing of calibrations can also impact on accuracy. The standard approach suggests calibration every 12 h, using a blood glucose sample. Calibration of the CGM should ideally be done during euglycaemia, with stable glucose readings. This is to avoid calibration errors due to the diffusional time lag. However, there is a tendency in clinical practice to undertake calibrations when there is a clinical indication to take a blood glucose level, typically during a hypoglycaemic event. Tiberi et al. (45) compared calibration twice a day vs. three times a day, but this did not improve sensor accuracy. This lack of effect of additional calibrations could be explained by the calibration algorithms used, whereby each blood glucose sample informs the CGM value for the next 24 h. Furthermore, the devices used as the comparative “gold standard” for measurement of blood glucose on neonatal units all have their own limitations of accuracy, depending on the device model and methodology (46).

A number of more recent CGM models have factory calibration settings so do not require blood glucose being taken for calibration. This includes the stand-alone non-adjunctive factory-calibrated FreeStyle Libre® (Abbott Diabetes Care, Alameda, CA, USA). The accuracy of the FreeStyle Libre® was evaluated in neonates, and the CGM values were significantly higher particularly in the first 3 h post-insertion. However, only 37 pairs of glucose values were available, and the MARD score was not reported (47). Assuming that the accuracy of factory calibrated CGM systems can be improved and validated for the neonate, they would be preferred, as they would reduce the frequency of blood samples and the workload of the clinical team. There are other factors that can lead to reduced point accuracy over time including zero-mean error and drift but how developmental maturity impacts on this has not been studied.

Having a low point accuracy can also lead to an erroneous calculation of sensitivity for detecting hypoglycaemia. The MARD score for hypoglycaemic values (2.8–3.9 mmol/L, 50–70 mg/dl) is often higher than in euglycaemic values in patients with diabetes (48, 49). A recent systematic review concluded that the sensitivity of CGM for detecting hypoglycaemia was low (34). This may partly be due to the increased rate of change during episodes of hypoglycaemia. Plus, any calibration errors will have a greater relative effect for low glucose concentrations. Hence, detection of hypoglycaemia using CGM should not rely on single comparative values. Point accuracy is important; however, such assessment does not address the potential clinical value of CGM in the setting of intensive care: to provide trend information, rather than diagnostic accuracy. Attempts have been made to define methodologies to characterize the clinical safety/utility in the context of trend accuracy analyses, using measures similar to the error grid plots used for point of care devices, but these have not been widely adopted as they are complex models and difficult to apply in clinical practice (50).

### Long term outcomes

Although studies have shown the association between clinically silent hypoglycaemia and worse neurodevelopment outcomes, and the ability of CGM to reduce the risk of hypoglycaemia there are currently no interventional CGM studies with long term neurodevelopmental follow up. Current interventional studies are limited by their size (51). Larger interventional studies will be critically important in determining the overall health benefits of CGM in the range of neonates at risk from hypoglycaemia.

## The future use of CGM in neonatal intensive care

Clinical studies have shown clear benefits in detecting and targeting glucose levels in neonates most at risk from hypoglycaemia, but appropriately powered studies are required to demonstrate long term clinical benefits. Follow up of cohorts of children who were managed with CGM as neonates such as the NIRTURE and REACT trials would be valuable in providing data on long term impact (4, 52). With unbiased data collected every 5 min CGM is an important tool in addressing the controversies over the optimal targets of glucose levels in neonates and how to achieve them. A number of centres with experience of CGM in the neonate are now using CGM in specific patients, such as extremely preterm neonates and those with persistent hypoglycaemia. However, it is important that more studies are undertaken to establish impact on long term clinical outcomes. Development of devices for the specific needs of the neonate, including insertion methods and accuracy at low glucose thresholds, would facilitate further studies to determine their optimal clinical use and facilitate adoption into clinical practice.

## Key messages

### What are the practical considerations for clinical use?

1. They are not designed or licensed for use in the neonate
2. The benefits relate to information on trends over time
3. They are not accurate enough for diagnostic testing
4. They are currently an adjunct to blood glucose monitoring not a replacement

### Which neonates may benefit?

1. Extreme preterm neonates with dysglycaemia
2. Neonates on insulin treatment
3. Neonates with persistent hypoglycaemia (congenital hyperinsulinism, growth restricted neonates)

## References

[1] National Institute for Health and Care Excellence. Type 1 diabetes in adults: diagnosis and management (NG17). (2022). Available at: https://www.nice.org.uk/guidance/ng17 (cited 2022 May 24). 32017485

[2] Hubbard EM Hay WW. The term newborn: hypoglycemia. Clin Perinatol. (2021) 48(3):665–79. 10.1016/j.clp.2021.05.013 34353586

[3] Cornblath M Ichord R. Hypoglycemia in the neonate. Semin Perinatol. (2000) 24(2):136–49. 10.1053/sp.2000.6364 10805169

[4] Beardsall K Thomson L Guy C Iglesias-Platas I van Weissenbruch MM Bond S Real-time continuous glucose monitoring in preterm infants (REACT): an international, open-label, randomised controlled trial. Lancet Child Adolesc Health. (2021) 5(4):265–73. 10.1016/S2352-4642(20)30367-9 33577770 PMC7970623

[5] Mola-Schenzle E Staffler A Klemme M Pellegrini F Molinaro G Parhofer KG Clinically stable very low birthweight infants are at risk for recurrent tissue glucose fluctuations even after fully established enteral nutrition. Arch Dis Child Fetal Neonatal Ed. (2015) 100(2):F126–131. 10.1136/archdischild-2014-306168 25381093

[6] Harris DL Weston PJ Signal M Chase JG Harding JE. Dextrose gel for neonatal hypoglycaemia (the sugar babies study): a randomised, double-blind, placebo-controlled trial. Lancet Lond Engl. (2013) 382(9910):2077–83. 10.1016/S0140-6736(13)61645-1 24075361

[7] Harris DL Weston PJ Gamble GD Harding JE. Glucose profiles in healthy term infants in the first 5 days: the glucose in well babies (GLOW) study. J Pediatr. (2020) 223:34–41. 10.1016/j.jpeds.2020.02.079 32381469

[8] McKinlay CJD Alsweiler JM Anstice NS Burakevych N Chakraborty A Chase JG Association of neonatal glycemia with neurodevelopmental outcomes at 4.5 years. JAMA Pediatr. (2017) 171(10):972–83. 10.1001/jamapediatrics.2017.1579 28783802 PMC5710616

[9] Perri A Giordano L Corsello M Priolo F Vento G Zecca E Continuous glucose monitoring (CGM) in very low birth weight newborns needing parenteral nutrition: validation and glycemic percentiles. Ital J Pediatr. (2018) 44(1):1–6. 10.1186/s13052-018-0542-5 30134937 PMC6106728

[10] Uettwiller F Chemin A Bonnemaison E Favrais G Saliba E Labarthe F. Real-time continuous glucose monitoring reduces the duration of hypoglycemia episodes: a randomized trial in very low birth weight neonates. Plos One. (2015) 10(1):e0116255. 10.1371/journal.pone.0116255 25590334 PMC4295867

[11] Galderisi A Facchinetti A Steil GM Ortiz-Rubio P Cavallin F Tamborlane W V Continuous glucose monitoring in very preterm infants: a randomized controlled trial. Pediatrics. (2017) 140(4):e20171162. 10.1542/peds.2017-1162 28916591

[12] Tabery K Černý M Urbaniec K Vaniš M Zoban P Štechová K. Continuous glucose monitoring as a screening tool for neonatal hypoglycemia in infants of diabetic mothers. J Matern Fetal Neonatal Med. (2020) 33(11):1889–94. 10.1080/14767058.2018.1533941 30570366

[13] Nally LM Bondy N Doiev J Buckingham BA Wilson DM. A feasibility study to detect neonatal hypoglycemia in infants of diabetic mothers using real-time continuous glucose monitoring. Diabetes Technol Ther. (2019) 21(4):170–6. 10.1089/dia.2018.0337 30839229

[14] Braune K Wäldchen M Raile K Hahn S Ubben T Römer S Open-source technology for real-time continuous glucose monitoring in the neonatal intensive care unit: case study in a neonate with transient congenital hyperinsulinism. J Med Internet Res. (2020) 22(12):e21770. 10.2196/21770 33275114 PMC7748959

[15] Win M Beckett R Thomson L Thankamony A Beardsall K. Continuous glucose monitoring in the management of neonates with persistent hypoglycemia and congenital hyperinsulinism. J Clin Endocrinol Metab. (2022) 107(1):E246–53. 10.1210/clinem/dgab601 34407200 PMC8830056

[16] Montaldo P Caredda E Pugliese U Zanfardino A Delehaye C Inserra E Continuous glucose monitoring profile during therapeutic hypothermia in encephalopathic infants with unfavorable outcome. Pediatr Res. (2020) 88(2):218–24. 10.1038/s41390-020-0827-4 32120381

[17] Beardsall K Ogilvy-Stuart a L Ahluwalia J Thompson M Dunger DB. The continuous glucose monitoring sensor in neonatal intensive care. Arch Dis Child Fetal Neonatal Ed. (2005) 90(4):F307–FF310. 10.1136/adc.2004.051979 16036889 PMC1721924

[18] Szymońska I Jagła M Starzec K Kwinta P. Glycemic variability in continuous glucose monitoring negatively correlates with gestational age in very low birth weight infants. J Matern Fetal Neonatal Med. (2020) 33(17):3041–3. 10.1080/14767058.2019.1566313 30614329

[19] Beardsall K Thomson L Elleri D Dunger DB Hovorka R. Feasibility of automated insulin delivery guided by continuous glucose monitoring in preterm infants. Arch Dis Child Fetal Neonatal Ed. (2020) 105(3):F279–84. 10.1136/archdischild-2019-316871 PMC7363782 31399480

[20] Zhou T Dickson J Shaw G Chase J. Continuous glucose monitoring measures can be used for glycemic control in the icu: an in-silico study. J Diabetes Sci Technol. (2018) 12(1):7–19. 10.1177/1932296817738791 29103302 PMC5761989

[21] Weber A Harrison TM. Reducing toxic stress in the neonatal intensive care unit to improve infant outcomes. Nurs Outlook. (2019) 67(2):169–89. 10.1016/j.outlook.2018.11.002 30611546 PMC6450772

[22] Campbell-Yeo M Eriksson M Benoit B. Assessment and management of pain in preterm infants: a practice update. Children. (2022) 9(2):244. 10.3390/children9020244 35204964 PMC8869922

[23] Thomson L Elleri D Bond S Howlett J Dunger DB Beardsall K. Targeting glucose control in preterm infants: pilot studies of continuous glucose monitoring. Arch Dis Child Fetal Neonatal Ed. (2019) 104(4):F353–9. 10.1136/archdischild-2018-314814 30232094 PMC6764251

[24] Perri A Tiberi E Giordano L Sbordone A Patti ML Iannotta R Strict glycaemic control in very low birthweight infants using a continuous glucose monitoring system: a randomised controlled trial. Arch Dis Child Fetal Neonatal Ed. (2022) 107(1):26–31. 10.1136/archdischild-2020-320540 34039690

[25] Petrou S Kim S Bond S Allison A Beardsall K. Cost-effectiveness of real time continuous glucose monitoring to target glucose control in preterm infants. Semin Perinatol. (2021) 45(3):151392. 10.1016/j.semperi.2021.151392 33549333

[26] Saha P Beardsall K. Perioperative continuous glucose monitoring in a preterm infant. BMJ Case Rep. (2018) 2018:1–3. 10.1136/bcr-2018-224728 PMC6040521 29950500

[27] Pertierra-cortada A Ramon-krauel M Iriondo-Sanz M Iglesias-platas I. Instability of glucose values in very preterm babies at term postmenstrual age. J Pediatr. (2014) 165:1146–53. 10.1016/j.jpeds.2014.08.029 25260622

[28] Kamino D Almazrooei A Pang EW Widjaja E Moore AM Chau V Abnormalities in evoked potentials associated with abnormal glycemia and brain injury in neonatal hypoxic-ischemic encephalopathy. Clin Neurophysiol. (2021) 132(1):307–13. 10.1016/j.clinph.2020.09.024 33158762 PMC7855101

[29] Pinchefsky EF Hahn CD Kamino D Chau V Brant R Moore AM Hyperglycemia and glucose variability are associated with worse brain function and seizures in neonatal encephalopathy: a prospective cohort study. J Pediatr. (2019) 209:23–32. 10.1016/j.jpeds.2019.02.027 30982528

[30] Tam EWY Kamino D Shatil AS Chau V Moore AM Brant R Hyperglycemia associated with acute brain injury in neonatal encephalopathy. NeuroImage Clin. (2021) 32:102835. 10.1016/j.nicl.2021.102835 34601311 PMC8496301

[31] Kalogeropoulou MS Thomson L Beardsall K. Continuous glucose monitoring during therapeutic hypothermia for hypoxic ischaemic encephalopathy: a feasibility study. Arch Dis Child Fetal Neonatal Ed. (2022). fetalneonatal–2022-324593. 10.1136/archdischild-2022-324593 36600516

[32] Stewart ZA Thomson L Murphy HR Beardsall K. A feasibility study of paired continuous glucose monitoring intrapartum and in the newborn in pregnancies complicated by type 1 diabetes. Diabetes Technol Ther. (2019) 21(1):20–7. 10.1089/dia.2018.0221 30620640

[33] Shah R Dai DWT Alsweiler JM Brown GTL Chase JG Gamble GD Association of neonatal hypoglycemia with academic performance in mid-childhood. JAMA. (2022) 327(12):1158–70. 10.1001/jama.2022.0992 35315886 PMC8941348

[34] Nava C Modiano Hedenmalm A Borys F Hooft L Bruschettini M Jenniskens K. Accuracy of continuous glucose monitoring in preterm infants: a systematic review and meta-analysis. BMJ Open. (2020) 10(12):1–9. 10.1136/bmjopen-2020-045335 33361084 PMC7768969

[35] Geoffrey M Brazg R Richard W. Freestyle navigator continuous glucose monitoring system with TRUstart algorithm, a 1-hour warm-up time. J Diabetes Sci Technol. (2011) 5(1):99–106. 10.1177/193229681100500114 21303631 PMC3045239

[36] Zisser HC Bailey TS Schwartz S Ratner RE Wise J. Accuracy of the SEVEN® continuous glucose monitoring system: comparison with frequently sampled venous glucose measurements. J Diabetes Sci Technol. (2009) 3(5):1146–54. 10.1177/193229680900300519 20144429 PMC2769895

[37] Bailey TS Ahmann A Brazg R Christiansen M Garg S Watkins E Accuracy and acceptability of the 6-day enlite continuous subcutaneous glucose sensor. Diabetes Technol Ther. (2014) 16(5):277–83. 10.1089/dia.2013.0222 24758729

[38] Klueh U Liu Z Feldman B Henning TP Cho B Ouyang T Metabolic biofouling of glucose sensors in vivo: role of tissue microhemorrhages. J Diabetes Sci Technol. (2011) 5(3):583–95. 10.1177/193229681100500313 21722574 PMC3192625

[39] Gerritsen M Jansen JA Kros A Vriezema DM Sommerdijk NA Nolte RJ Influence of inflammatory cells and serum on the performance of implantable glucose sensors. J Biomed Mater Res. (2001) 54(1):69–75. 10.1002/1097-4636(200101)54:1<69::AID-JBM8>3.0.CO;2-Q 11077404

[40] Freckmann G Pleus S Grady M Setford S Levy B. Measures of accuracy for continuous glucose monitoring and blood glucose monitoring devices. J Diabetes Sci Technol. (2019) 13(3):575–83. 10.1177/1932296818812062 30453761 PMC6501529

[41] Wackernagel D Dube M Blennow M Tindberg Y. Continuous subcutaneous glucose monitoring is accurate in term and near-term infants at risk of hypoglycaemia. Acta Paediatr Int J Paediatr. (2016) 105(8):917–23. 10.1111/apa.13479 27203555

[42] Cengiz E Tamborlane WV. A tale of two compartments: interstitial versus blood glucose monitoring. Diabetes Technol Ther. (2009) 11(S1):2–3. 10.1089/dia.2009.0002 PMC2903977 19469670

[43] Shi T Li D Li G Zhang Y Xu K Lu L. Modeling and measurement of correlation between blood and interstitial glucose changes. J Diabetes Res. (2016) 2016:1–10. 10.1155/2016/4596316 PMC4863111 27239479

[44] Alva S Bailey T Brazg R Budiman ES Castorino K Christiansen MP Accuracy of a 14-day factory-calibrated continuous glucose monitoring system with advanced algorithm in pediatric and adult population with diabetes. J Diabetes Sci Technol. (2022) 16(1):70–7. 10.1177/1932296820958754 32954812 PMC8875061

[45] Tiberi E Cota F Barone G Perri A Romano V Iannotta R Continuous glucose monitoring in preterm infants: evaluation by a modified clarke error grid. Ital J Pediatr. (2016) 42(1):1–7. 10.1186/s13052-016-0236-9 26960676 PMC4784331

[46] Ba Y Xu J Yuan L Zhu H Yang Y Lam MM Assessment of the performance of blood glucose monitoring systems for monitoring dysglycaemia in neonatal patients. BMJ Paediatr Open. (2018) 2(1):1–7. 10.1136/bmjpo-2018-000339 30397671 PMC6203032

[47] Nishimura E Oka S Ozawa J Tanaka K Momose T Kabe K Safety and feasibility of a factory-calibrated continuous glucose monitoring system in term and near-term infants at risk of hypoglycemia. Turk Arch Pediatr. (2021) 56(2):115–20. 10.5152/TurkArchPediatr.2020.20183 34286319 PMC8269939

[48] Davis GM Spanakis EK Migdal AL Singh LG Albury B Urrutia MA Accuracy of dexcom G6 continuous glucose monitoring in non–critically ill hospitalized patients with diabetes. Diabetes Care. (2021) 44(7):1641–6. 10.2337/dc20-2856 34099515 PMC8323182

[49] Moser O Pandis M Aberer F Kojzar H Hochfellner D Elsayed H A head-to-head comparison of personal and professional continuous glucose monitoring systems in people with type 1 diabetes: hypoglycaemia remains the weak spot. Diabetes Obes Metab. (2019) 21(4):1043–8. 10.1111/dom.13598 30484947 PMC6590188

[50] Signal M Gottlieb R LeCompte A Chase J. Continuous glucose monitoring and trend accuracy: news about a trend compass. J Diabetes Sci Technol. (2014) 8(5):986–97. 10.1177/1932296814533091 24876437 PMC4455373

[51] Galderisi A. Impact of continuous glucose monitoring on cerebral oxygenation in preterm infants (the babyglucolight trial). clinicaltrials.gov; (2020). Report No.: NCT04347590. Available at https://clinicaltrials.gov/ct2/show/NCT04347590 (cited 2023 Feb 9).

[52] Beardsall K Vanhaesebrouck S Ogilvy-Stuart AL Vanhole C Palmer CR van Weissenbruch M Early insulin therapy in very-low-birth-weight infants. N Engl J Med. (2008) 359(18):1873–84. 10.1056/NEJMoa0803725 18971490

